# A multi-layer monitoring system for clinical management of Congestive Heart Failure

**DOI:** 10.1186/1472-6947-15-S3-S5

**Published:** 2015-09-04

**Authors:** Gabriele Guidi, Luca Pollonini, Clifford C Dacso, Ernesto Iadanza

**Affiliations:** 1 Department of Information Engineering, Università degli Studi di Firenze, Via di S. Marta 3, Florence, 50139, Italy; 2 ICON Foundation, Via Nello Carrara 1, Sesto Fiorentino, 50019, Italy; 3 Department of Engineering Technology, University of Houston -- 300 Technology Building, Houston TX 77204, USA; 4 Abramson Center for the Future of Health, University of Houston -- 300 Technology Building, Houston TX 77204, USA; 5 Department of Molecular and Cellular Biology, Baylor College of Medicine, One Baylor Plaza, Houston TX 77030, USA

## Abstract

**Background:**

Congestive Heart Failure (CHF) is a serious cardiac condition that brings high risks of urgent hospitalization and death. Remote monitoring systems are well-suited to managing patients suffering from CHF, and can reduce deaths and re-hospitalizations, as shown by the literature, including multiple systematic reviews.

**Methods:**

The monitoring system proposed in this paper aims at helping CHF stakeholders make appropriate decisions in managing the disease and preventing cardiac events, such as decompensation, which can lead to hospitalization or death. Monitoring activities are stratified into three layers: scheduled visits to a hospital following up on a cardiac event, home monitoring visits by nurses, and patient's self-monitoring performed at home using specialized equipment. Appropriate hardware, desktop and mobile software applications were developed to enable a patient's monitoring by all stakeholders. For the first two layers, we designed and implemented a Decision Support System (DSS) using machine learning (Random Forest algorithm) to predict the number of decompensations per year and to assess the heart failure severity based on a variety of clinical data. For the third layer, custom-designed sensors (the Blue Scale system) for electrocardiogram (EKG), pulse transit times, bio-impedance and weight allowed frequent collection of CHF-related data in the comfort of the patient's home.

We also performed a short-term Heart Rate Variability (HRV) analysis on electrocardiograms self-acquired by 15 healthy volunteers and compared the obtained parameters with those of 15 CHF patients from PhysioNet's PhysioBank archives.

**Results:**

We report numerical performances of the DSS, calculated as multiclass accuracy, sensitivity and specificity in a 10-fold cross-validation. The obtained average accuracies are: 71.9% in predicting the number of decompensations and 81.3% in severity assessment. The most serious class in severity assessment is detected with good sensitivity and specificity (0.87 / 0.95), while, in predicting decompensation, high specificity combined with good sensitivity prevents false alarms. The HRV parameters extracted from the self-measured EKG using the Blue Scale system of sensors are comparable with those reported in the literature about healthy people.

**Conclusions:**

The performance of DSSs trained with new patients confirmed the results of previous work, and emphasizes the strong correlation between some CHF markers, such as brain natriuretic peptide (BNP) and ejection fraction (EF), with the outputs of interest. Comparing HRV parameters from healthy volunteers with HRV parameters obtained from PhysioBank archives, we confirm the literature that considers the HRV a promising method for distinguishing healthy from CHF patients.

## Background

Congestive Heart Failure (CHF or HF) is a serious cardiac condition that carries high risks of emergency hospitalization and death. CHF is prevalent in the aging population, as it affects 3-20 out of 1,000 adults and up to 10% of people aged between 80 and 89. In the UK, CHF consumes almost 2% of the National Health Service's budget, most of the cost being linked to hospital admissions [1].

Drug therapy is the mainstay of treatment for CHF. However, management has evolved over the last several years from a traditional model almost solely based on crisis intervention towards more proactive and preventative disease management models supported by a combination of medications and preventive paradigms, including a healthy lifestyle. This management concept, defined as Chronic Care Model (CCM) [2], aims at establishing the pillars of a "medicine of initiative" in which the physician takes action before the disease worsens, as opposed to the old model of "waiting medicine" in which the patient is treated when the disease is already in its acute phase. The ultimate goal of CCM is to reduce re-hospitalizations that have negative effects on both the quality of life of the patient and the national annual cost for treating CHF. Therefore, identifying the causes that lead to CHF-related re-hospitalization provides the opportunity to redesign care to prevent re-hospitalization and subsequently to improve quality of life [3].

Building an effective disease management strategy requires analyzing many variables, including the care setting, the ability of the patient and family to perform self-management and the severity of the disease [3]. A Cochrane Collaboration review found that home monitoring (by telephone support and vital sign monitoring) significantly reduces all causes of mortality in CHF patients [4]. A more recent review by the Cochrane Collaboration [1] concluded that follow-up on CHF patients through periodic telephone calls and home visits resulted in fewer deaths from all CHF causes compared to periodic, scheduled visits to a CHF specialist or hospital or to multidisciplinary interventions carried out by a team of professionals helping the transition from the hospital to the home, although the study was not sufficiently comprehensive to determine the best overall strategy. In addition, current practice guidelines and consensus statements on CHF, including the most recent American College of Cardiology/American Heart Failure guideline (2013), agree that CHF is a multifactorial disease that requires continuity of care outside the hospital, including coordination of multiple health professions and proactive self-management by the patient [5].

However, the intrinsic complexity of proactive self-management, mainly due to detailed and nuanced protocols, is a significant cause for lack of adherence by patients and their families, which in turn results in high rates of unnecessary hospitalizations [6], [7]. Decision support systems based on personalized, actionable patient data can facilitate communication and collaboration across care levels and therefore represent an important advancement towards the solution of this issue. In this work, we propose a solution consisting of a combination of custom-designed sensors for home measurements with a three-layer monitoring model (two clinical layers and one patient layer) that involves clinical stakeholders assisted by two Decision Support Systems (DSS) based on a powerful machine learning engine. Desktop and mobile software tools complement the system by offering a friendly interface for caregivers. The novelty of this work compared to the state of the art is the application of DSSs designed specifically for each clinical stakeholder (nurses and physicians), together with the monitoring model based on three layers that allows an effective compromise between the quality of the monitored parameters (and the possibility of acquiring parameters that are CHF markers) and their acquisition frequency. In previous studies, we identified and implemented the appropriate machine learning technique for dealing with the CHF. In this work, we retrained the system with larger, more structured datasets in order to improve prediction performances and the statistical validity of the results. Another innovative aspect of this work is the integration of several independent modules (DSSs, sensor devices for patient homecare, telemedicine infrastructures, cross-platform interfaces) into a single system generating new workflows that will allow stakeholders at all levels to take clinical decisions in a multifactorial manner.

## Methods

### Design concept

**Figure 1:**
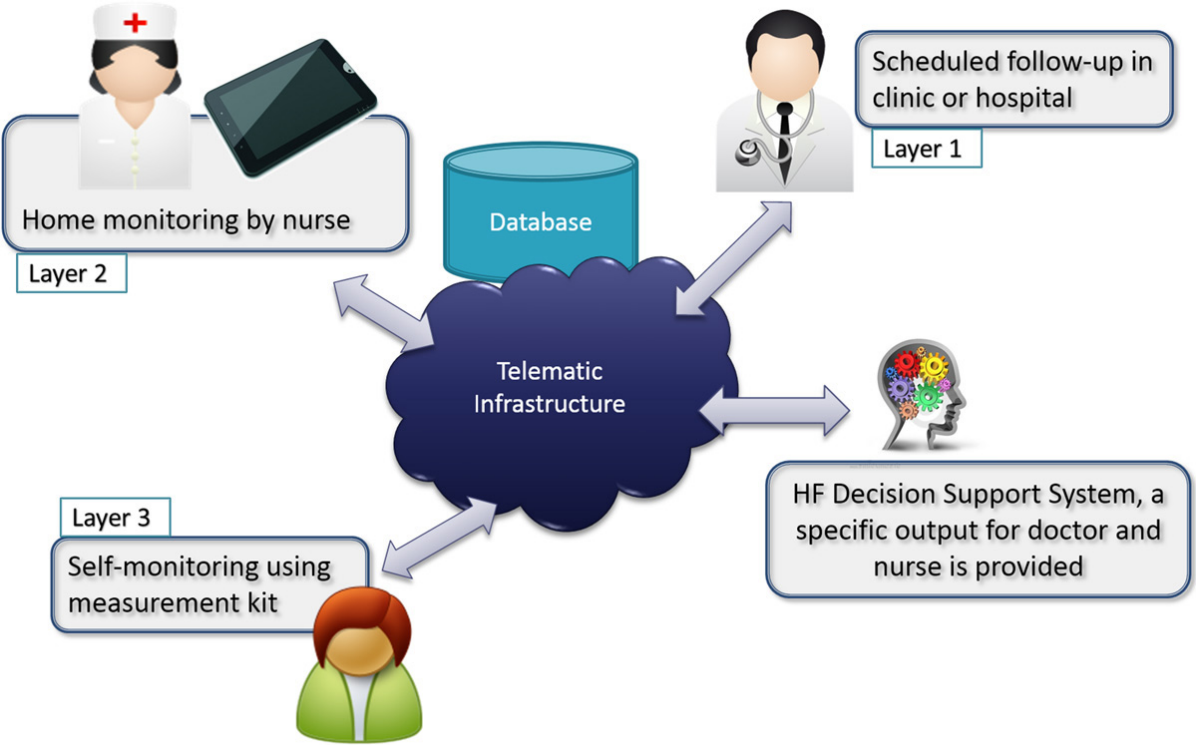
**Monitoring Schema and respective stakeholders**.

The system described in this paper aims to help CHF stakeholders (families, patients, and caregivers at all levels) make appropriate CHF management decisions through a three-layer monitoring system (which partly reflects the solution described in [1]) consisting of two clinical layers (Layers 1 and 2) and one patient layer (Layer 3). The system relies on the concept of collaborative framework and is based on an HIPAA-compliant cloud architecture that allows centralized collection and secure sharing of information among different layers and stakeholders (see Figure 1). In this context, clinical stakeholders in the upper two layers are provided with a Decision Support System (DSS) geared to specific aspects of CHF, whereas Layer 3 is currently focused on data collection in the patient's home. In this work, we integrated CHF special-purpose modules which were tested in our previous work [8] with other modules appositely designed for testing the proposed monitoring and decision support system as a whole. When compared to individual modules taken separately, our proposed solution results in information and decision support being provided to stakeholders in a more multifactorial and multi-parametric manner, while reducing the time needed for patient evaluation and the cost of both false positives (unnecessary hospital readmissions) and false negatives (missed necessary readmissions).

Based on a collaborative investigation involving expert clinicians that modeled the time progression of CHF, we proposed a system including several types of monitoring scenarios differing by the number of clinical measures and their sampling frequency [9]. Specifically, we designed a multi-layer monitoring structure in which a set of measured clinical parameters were weighted with inverse proportion to the frequency with which they are acquired, so as to establish a reasonable trade-off between the number of useful clinical data points and the burden and cost of collecting them.

The most comprehensive, yet least frequent, monitoring layer (Layer 1) consists of a complete scheduled cardiac outpatient visit to be performed in a hospital or at another point of care. In addition to a thorough physical exam and collection of medical history, this visit allows the recording of all clinical measures known to be relevant to CHF, including those that vary slowly over the progression of the disease such as ejection fraction (EF) and Brain Natriuretic Peptide (BNP). Pharmaceutical therapy is also prescribed as the doctor sees fit. This layer is characterized by an acquisition rate of clinical parameters of about 6 months, corresponding to the frequency of the comprehensive visit. Through a dedicated desktop application described in [10], the specialist can keep the clinical status of the patient up to date with newly acquired parameters, therapy, medical history and model-based prognosis scores, all of which are stored into a database shared across all layers of the proposed system.

The second layer of monitoring (Layer 2) in order of acquisition frequency (every 1-2 weeks) is performed by a nurse visiting the patient at home using a measuring kit coupled with a tablet computer. A dedicated app (described in the section below) allows the nurse to enter all relevant measures (i.e., clinical parameters and questionnaires) that are subsequently stored into the shared database system.

While Layers 1 and 2 described above are designed around caregivers (doctor and nurses, respectively) who are expert in CHF, Layer 3 is entirely patient-oriented. In this layer, the CHF patient is entrusted with the responsibility of contributing to his/her own care by actively engaging in disease self-management, as proposed in the CCM model [2]. To achieve this, monitoring in Layer 3 consists of a frequent data acquisition (1-2 times/day) of several CHF-related parameters such as electrocardiogram (EKG), heart rate (HR), pulse transit times (PTT), weight and bioimpedance (bioZ) performed at home using custom-designed monitoring devices and shared across layers.

**Figure 2:**
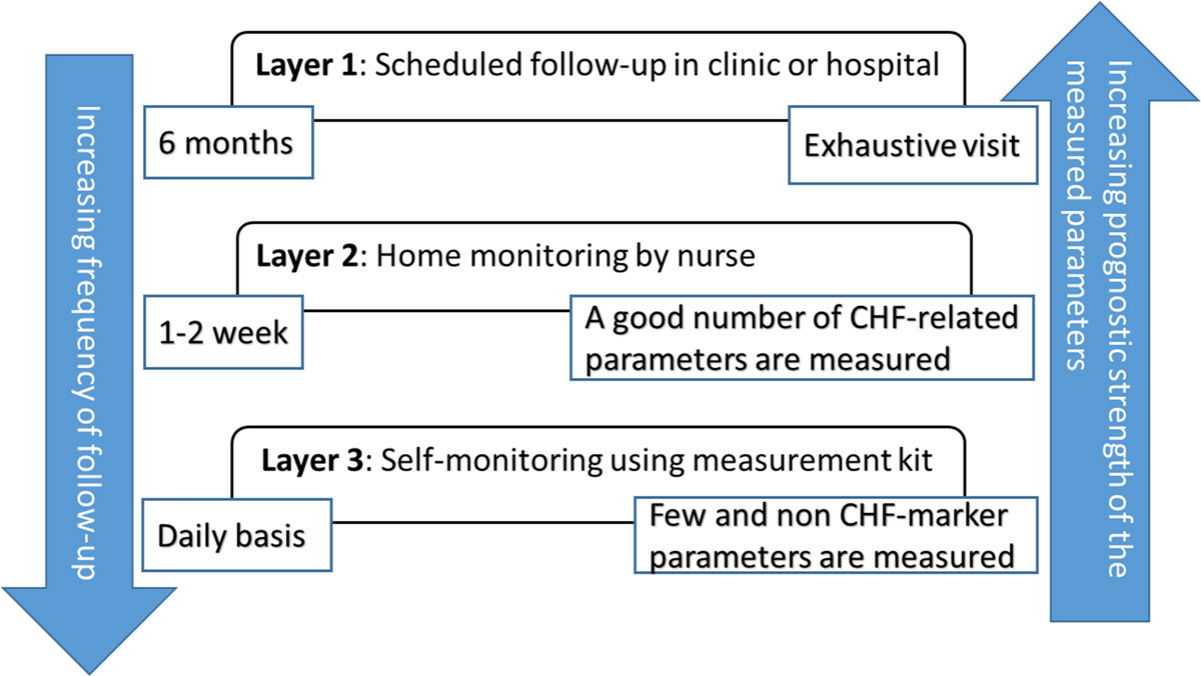
**The layers of monitoring: compromise between the follow-up frequency and the prognostic strength of the measured parameters**.

In this framework, no layer of the system operates independently from the other layers. At the first hospitalization for acute symptoms of CHF or on the first scheduled visit for evaluation of chronic symptoms, patients are enrolled in the various layers of monitoring depending on a calculated score that accounts for relevant indicators (e.g., age, comorbidities, number of past hospitalizations for heart disease, etc.), clinical data (BNP, EF, renal functions, etc.), as well as historical data and symptoms-based NYHA class [11]. This type of multi-layer monitoring leads to hierarchically structured time-dependent data; hence, an hypothetical patient who is under monitoring in all three layers will be exhaustively checked every 6 months in the hospital (collecting BNP, EF, 12-lead EKG, laboratory data, etc.), every 2 weeks by a nurse visit at home (consisting of physical examination and collecting capillary BNP data) and on a daily basis through self-measurements (weight, 2-lead EKG, PTT, bioZ). Figure 2 shows that such a complex system represents an effective tradeoff between the measurement frequency and the number and relevance of the parameters, since strong CHF markers (as found in [8]) require blood (BNP) or ultrasound (HF) testing that cannot be easily performed at home.

### Monitoring layers

#### Layer 1: Scheduled follow-up in clinic or hospital

The first layer of the proposed system provides the physician with a desktop application (previously described in [10]) with the following functions:

• Serves as a management software including CHF-specific computational and graphical tools for calculation of prognostic scores, smart management of therapy, display of various CHF-related parameters;

• Serves as an input portal for training of the machine-learning tool (DSS) used in Layer 2 (nurse visits);

• Displays management suggestions provided by the DSS underlying Layer 1;

• Acts as a control and display panel for Layer 3, self-monitoring performed autonomously by the patient at home.

The clinical parameters obtained and entered into the system by the physician in Layer 1 are:

• Height and weight (Body Mass Index)

• Systolic and diastolic blood pressure

• Heart rate

• Oxygen saturation

• Ejection fraction (EF)

• BNP or NT-proBNP

• Bioelectrical impedance vector (BIVA) parameters

• NYHA class

• 12-lead EKG report (e.g., presence of bundle branch block, tachycardia, atrial fibrillation, etc.)

• Etiology

• Comorbidity

• Current therapy, pharmaceutical and surgical (pacemaker or ICD ICD / CRT)

Previously released guidelines [5] indicate that Cardiac Troponin is an additional significant biomarker of CHF, although it is a more direct marker of myocardial infarction and acute coronary syndrome. However, we chose to include only one blood marker (BNP) that could be measured with portable point of care devices to minimize the cost of each measurement.

In this layer, prognostic scores such as 1-year and 5-year survival rates are calculated from patient-specific data using accepted models [10]. Layer 1's DSS provides the physician with a forecast of frequency of patient decompensation events to be expected during the subsequent year (e.g., none, 1-2 exacerbations, >2 exacerbations) using the predictive model described in [8].

#### Layer 2: Home monitoring by nurse

In this layer, data collection and monitoring is periodically performed by a nurse visiting the patient at home equipped with a set of portable devices. The measurement protocol includes:

• Examination of qualitative parameters (jugular turgor, skin color, ankle edema, pressure ulcer);

• Acquisition of vital signs: Weight, blood pressure, oxygen saturation, bioimpedance using portable instrument, capillary dosage of BNP using portable device.

**Figure 3:**
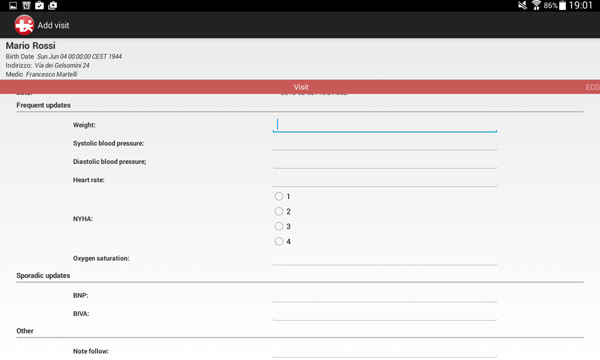
**Android app to enable nurses to acquire the parameters in the patient's home**.

To achieve time- and cost-effective monitoring, we designed an Android mobile application for tablets and smartphones that enables data collection and transmission to the shared database, so that data acquired at point of care are also immediately available to the cardiologist. Since it is impractical for the cardiologist to check clinical data on a daily basis, we trained the DSS in this monitoring layer to provide a stratification of the severity of the patient's condition in three levels (mild, moderate, severe) so to enable a closer follow-up on patients with higher risks of decompensation. Such classification is based on machine learning techniques described in [12] that take into account a multi-parametric description of the patient, rather than a threshold-based analysis of individual clinical measures. By mean of this app, the nurse is immediately alerted to the severity assessment of the patient's CHF and its variance from previous readings. A representative screenshot of the app used on a 10-inch tablet is shown in Figure 3.

#### Layer 3: Patient self-monitoring using measurement kit

In this monitoring layer, portable devices are provided to the patient for acquiring daily measurements of several physiological measures relevant to CHF. After collection, data are securely transmitted and stored in the HIPAA-compliant central database for immediate consultation and processing. Depending on the recommendation of the physician and the usage preference of the patient, the monitoring within Layer 3 will be performed choosing between devices with different form factors.

**Figure 4:**
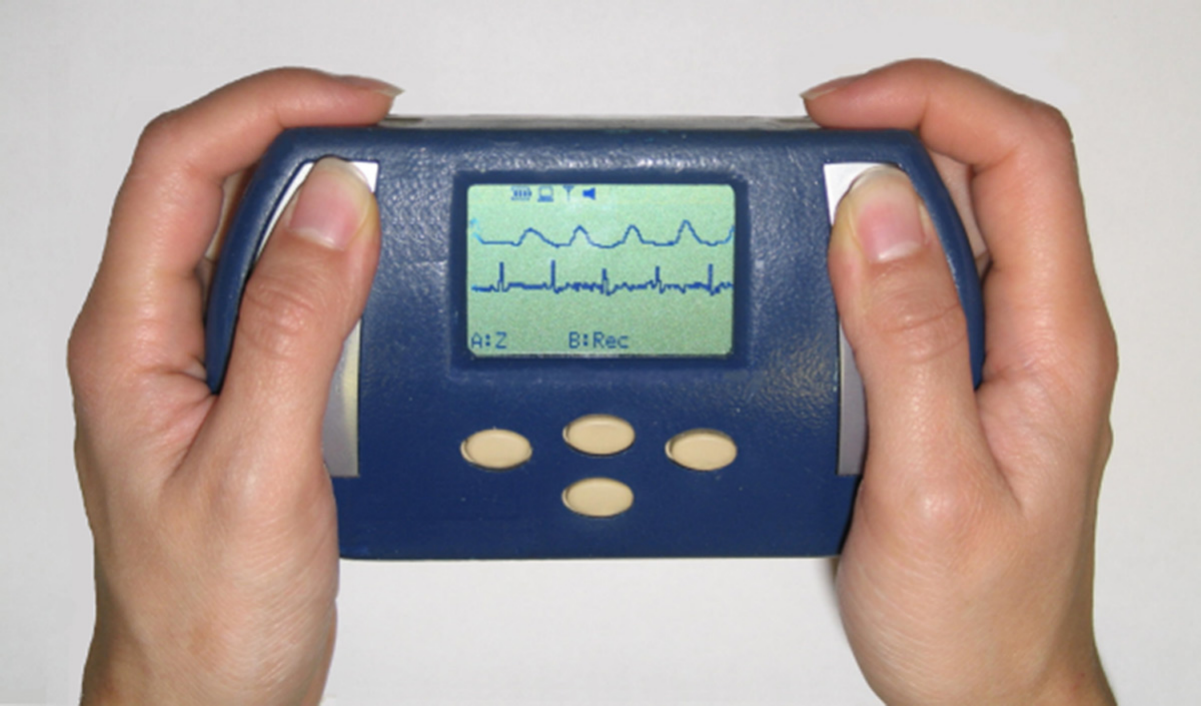
**Blue Box Device**.

One option is represented by a handheld device called BlueBox that integrates two sets of electrodes (left hand and right hand) for collecting 2-lead electrocardiography (EKG) and bio-impedance signals [13]. In addition, it embeds a photoplethysmography sensor (PPG) for the measurement of the pulse transit time (PTT), that is, the time interval between the cardiac contraction (EKG's R-wave) and the arrival of the blood wave to the periphery, i.e., the fingertip (Figure 4). PTT is a cardiovascular measure that has been shown to be related to arterial stiffness [14]. Recently, our group has also found that PTT is a valuable addition to heart rate as a surrogate of cardiac output, oxygen uptake and stroke volume during physical exercise [15]. Hence, PTT has the potential to provide additional quantitative information on the cardiovascular system without using measurement tools that are invasive or are available only in clinical settings. BlueBox communicates with the user by means of a small display and securely transmits the data to the central database using a Bluetooth gateway.

**Figure 5:**
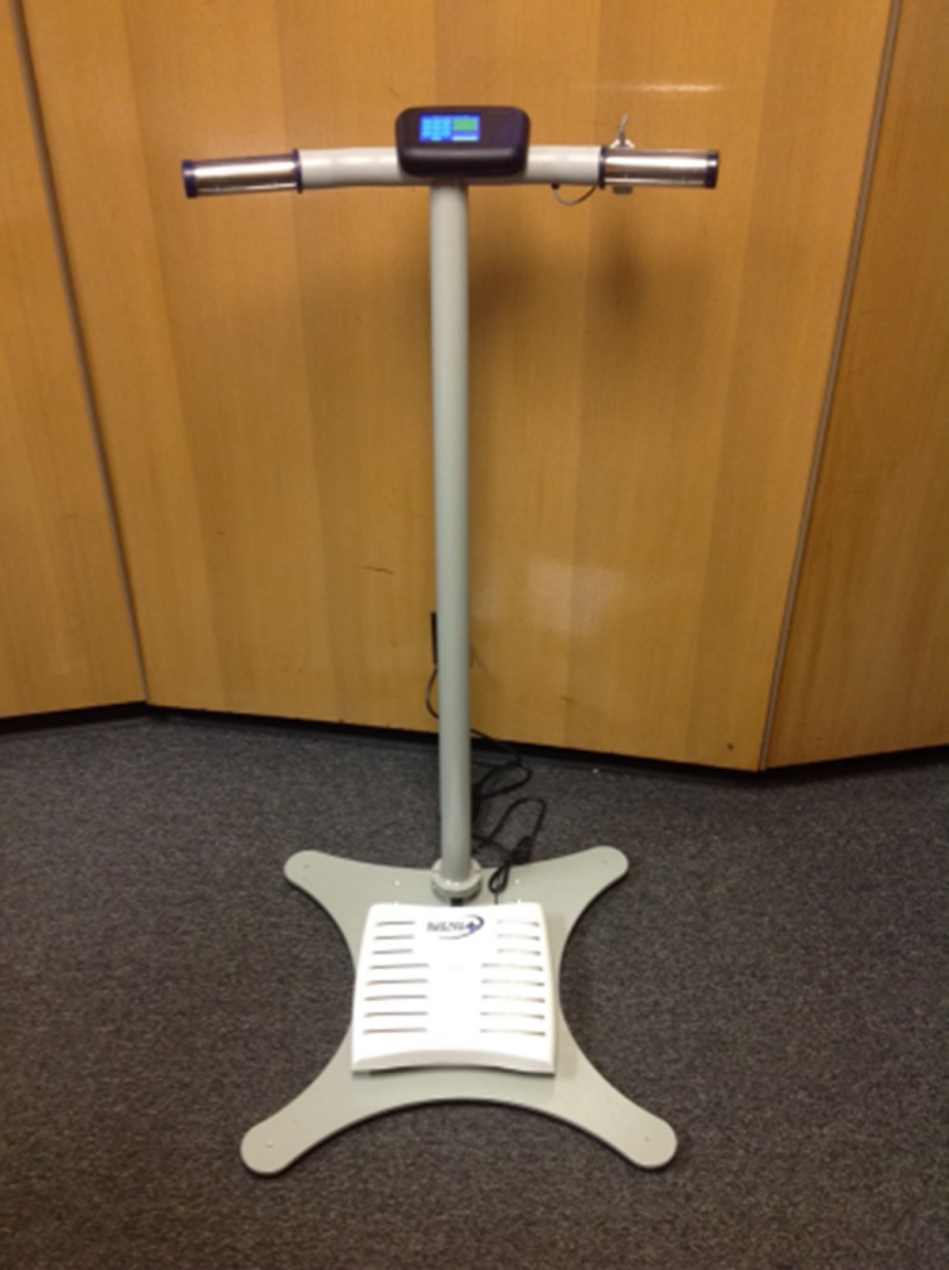
**Blue Scale Device**.

Surveys conducted on BlueBox users revealed that the majority of elderly CHF patients did not acquire a sufficient familiarity with the handheld device and would have preferred to deal with a tool that better fits their lifestyles. Hence, a second-generation device called BlueScale was conceived around a familiar household tool frequently used for CHF management: a bathroom scale. To expand the measurement capabilities of BlueBox and to meet this identified need, we modified a bathroom scale to integrate foot electrodes and attached a metal assembly consisting of a horizontal handlebar and a vertical post [16] integrating a pair of hand electrodes (one per hand), a finger-clipping PPG sensor and a large touchscreen display (Figure 5). In comparison to BlueBox, the improved design of BlueScale allowed the collection of a 3-lead EKG (left hand-right hand-left foot), whole-body bioimpedance and also weight, deviations in which over time are relevant to CHF. Data collected by BlueScale are transmitted to the central database via secure WiFi. Upon a preliminary adherence study, we found that this form factor was better adopted by the population of potential users [17].

In our proposed workflow model, decision-making is a responsibility that resides entirely with the caregivers and never relies on the patient's ability to interpret complex mechanisms and results. Since Layer 3 was designed solely to collect relevant data with high sampling frequency for upper layers where clinical decisions are made, this layer did not include any specific DSS.

### Cardiologist Dashboard

**Figure 6:**
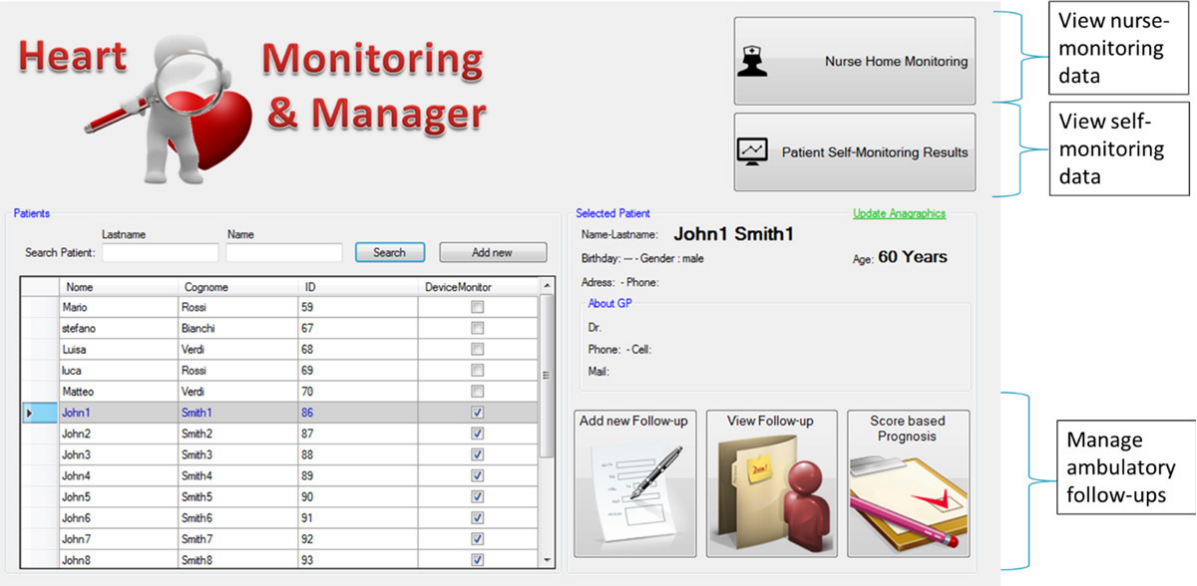
**Cardiologist Dashboard - main**.

**Figure 7:**
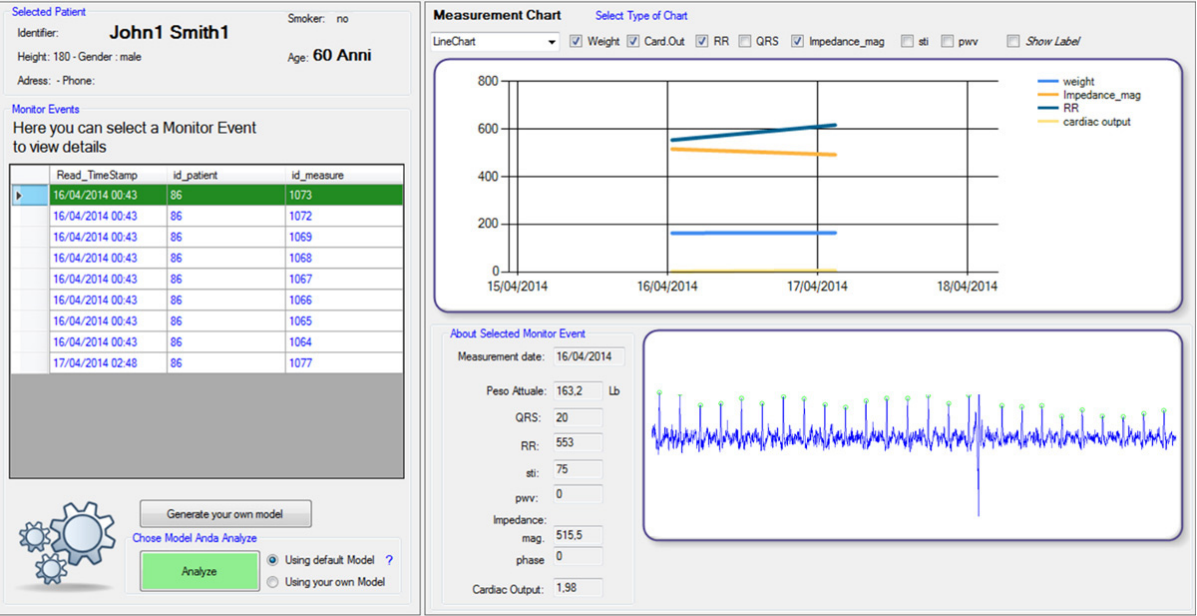
**Cardiologist Dashboard - Patient self-monitoring view**.

An important feature of the proposed system is the dashboard that collects and manages all the data incoming from the monitoring layers. The dashboard is integrated as a plug-in to the same experimental interface provided to the doctor for ambulatory follow-up monitoring (see Figure 6 and 7).

As can be seen in Figure 6, the cardiologist, after selecting a patient from the list, can access the management section of the ambulatory follow-up and can either enter data newly acquired in the hospital (Layer 1), or can display data coming from Layers 2 and 3 of monitoring (nurse or self-monitoring). In particular, Figure 7 shows the panel displaying data self-collected by the patient in addition to EKG-related parameters computed using the Heart Rate Variability Analysis Software tool (HRVAS) [18]. Studies show that HRV parameters are highly correlated with the severity of CHF and thus may be used as features to train machine learning algorithms aimed at predicting CHF decompensation [19,20]. Prior to display, the EKG waveform is processed using wavelet decomposition to eliminate some artifacts and to obtain a stable baseline (eliminating the effects of breathing and other fluctuations), whereas QRS complexes are detected using a template matching method.

## Experimental setup

The DSSs implemented in Layers 1 and 2 were trained and evaluated using internal anonymized datasets whereas publicly available CHF data (i.e., PhysioBank) and data acquired on healthy volunteers were used to evaluate the performance of Layer 3.

Unlike our previous work (see the results in [8]) where sparse, longitudinal datasets were used, we hereby utilized a cross-sectional dataset consisting of first-visit data of 250 patients. For DSS validation purposes, each record was retrospectively labeled by a cardiologist as:

a. *None, rare *or *frequent *according to the number of times the patient was hospitalized for CHF aggravation in the year after such first visit, corresponding respectively to never, <= 2 times, > 2 times

b. *Mild, moderate, or severe *according to the clinician's evaluation of the CHF status

After proper training, the DSS for Layer 1 should predict the frequency of CHF aggravation, whereas DSS for Layer 2 should automatically classify the severity of CHF using only clinical data collected during the patient's first visit.

Since subsets of clinical data were missing on some patients, not every numeric input could be used for training. Hence, we chose to test the DSS performance using the following 8 parameters, which were available for all 250 patients: systolic blood pressure (SBP), diastolic blood pressure (DBP), heart rate (HR), weight, BNP, ejection fraction (EF), gender, age. Table 1 summarizes the classification operated by the cardiologist based on his or her expertise.

**Table 1:** Dataset distribution.

Type of output	N° of patients in Class 1	N° of patients in Class 2	N° of patients in Class 3	Sum
CHF Severity (mild/moderate/severe)	93	92	65	250

CHF decompensation (none/rare/frequent)	161	55	64	250

Both DSSs for Layer 1 and Layer 2 were based on Random Forest [21], i.e., the machine learning technique that yielded the best results in our previous study [22] and in other classification-tree-based approaches [23]. In this work, we maintained the internal parameters of the Random Forest used previously [22] in order to test the ability of such a setting to perform on a different dataset. The parameters used in this study were:

• Number of trees in the forest = 2000, determined by evaluating the reduction of Out of Bag error by increasing the number of trees;

• Number of features to be used in each tree = 4, empirically determined in cross-validation in [22];

• Cut-off for each class was set in order to take into account the imbalance of the dataset. For CHF severity output it was (class 1:30 / class 2: 30 / class 3: 40) and for CHF decompensation (class 1: 50 / class 2: 20 / class 3: 30). Given that a low cut-off makes a class an "easy-winner" and observing Table 1, we see that these values are theoretically valid also for the new dataset. However, while as regards the severity assessment, these values are confirmed, with regard to the prediction of decompensation event, in this work we used a 70-15-15 configuration that takes into account the even more strong imbalance of the new dataset, in order to maximize the specificity of class 1 (have really no decompensation, either rare or frequent, if the system predicts "none"). This configuration was performed by observing the classes distribution in Table 1.

A meaningful internal parameter of the Random Forest training process is the mean decrease of the Gini impurity index, providing information on the importance that each input has on the prediction of the output value. In fact, the Gini impurity index is a measure of how often a randomly chosen element from the set would be incorrectly labeled if it were randomly labeled according to the distribution of labels in the subset. Breiman, the father of the Random Forest algorithm [21], proposed to evaluate the importance of a variable × for predicting Y in each tree of the forest by adding up the weighted impurity decreases and, in the most common implementations, the Gini impurity index was used. We used this index to evaluate whether our results reflect the known literature about CHF-marker parameters.

### Data evaluation and statistical analysis

To evaluate the performance of the DSS in Layers 1 and 2, we used a 10-fold cross-validation method. Since each DSS provided a three-class output, classification accuracy was measured using the multi-class formula:

$$Accuracy=\frac{\sum_{i=1}^{N^{\circ }Class}\frac{TP_{i}+TN_{i}}{TP_{i}+TN_{i}+FP_{i}+FN_{i}}}{N}$$

where TP, TN were the number of true positives and negatives, respectively, and FP, FN were the number of false positives and negatives, respectively, for each class.

In addition, we evaluated sensitivity and specificity of the three-way classification using the method "one class *versus *all the others", i.e., sensitivity and specificity were computed as in a binary classification predicting "severe vs. mild + moderate", "mild vs. moderate + severe" and "moderate vs. mild+severe". Classification and cross-validation using Random Forest were implemented in Matlab (The Mathworks, Natick, MA) with both homemade and GPLv3 license functions. We reported individual-fold and average results, as well as the number of critical errors, defined as cases in which a patient who had no decompensation (class *none*) or was in severity class *mild *was wrongly classified as one with *frequent *decompensation or as a *severe *CHF (critical error 1-3). Vice versa, critical errors 3-1 were defined as cases in which *frequent *decompensation or *severe *CHF were erroneously classified as *none *or *mild*, respectively.

For this study, data collected with self-monitoring devices (BlueBox and BlueScale) were available only on healthy patients [17]. However, we preliminarily assessed the potential of EKG-derived parameters to disambiguate healthy people from CHF patients using datasets freely available in literature. Specifically, we performed an analysis of short-term heart rate variability (HRV) using the HRV toolkit from PhysioNet [24] on 15 healthy patients with BlueScale in comparison to 15 CHF datasets obtained from PhysioBank. The HRV parameters yield by the toolkit were AVNN (average of all normal sinus to normal sinus (NN) intervals), SDNN (standard deviation of all NN intervals), RMSSD (square root of the mean of the sum of the squares of differences between adjacent NN intervals), pNN20, pNN50 (percentage of differences between adjacent NN intervals greater than 20 or 50 ms, respectively), TOTPWR (total spectral power of all NN intervals 0-0.4 Hz). All HRV parameters were measured in ms, except TOTPWR which was measured in ms^2^. Short-term HRV was performed on both cohorts after removing outlying data, following the guidelines provided with the analysis toolkit. Differences between healthy and CHF groups for all HRV parameters were statistically tested with Student's t-test with a significance level set at *p = *0.05. Experimental protocol and informed consent form were approved by the Institutional Review Board of participating institutions. Participant gave informant consent before being enrolled.

## Results

### Layer 1

**Figure 8:**
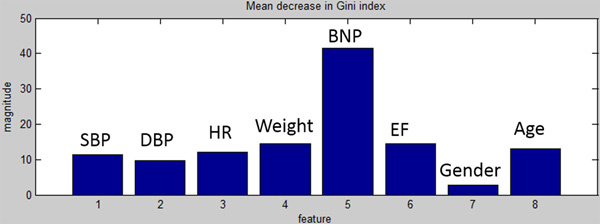
**Strong correlation between BNP (feature 5) and prediction of decompensation**.

The aim of the DSS in Layer 1 was to make a prediction of the frequency of CHF decompensation during the year after the first visit based on the snapshot of data available at such first visit. Table 2 shows the 10-fold cross-validation accuracy, sensitivity and specificity for one of the three classes (*none, rare *or *frequent*) evaluated against the other classes, repeated for all combinations of classes. Since there were no patients of class *frequent *in the seventh fold, the corresponding sensitivity was not indicated. On average, the accuracy of the classification was 72 ± 5 % (mean ± STD), whereas the average sensitivity and specificity computed across all comparisons were 60 ± 4 % and 78 ± 18 %, respectively. The table also reports the number of critical errors 1-3 (i.e., the DSS classified the patient as *none *instead of *frequent*) and of critical errors 3-1 (i.e., the DSS classified the patient as *frequent *instead of *none*). Out of 250 patients and cumulatively over the 10-fold validation, there were only 3 critical errors of type 1-3 and 2 critical errors of type 3-1. Figure 8 shows the mean decrease of the Gini index across the 8 selected features (SBP, DBP, HR, Weight, BNP, EF, Gender, Age), where BNP was found to be the strongest predictor.

**Table 2:** performances of DSS of layer 1.

Fold N°	Accuracy %	N° critical error 1-3	N° critical error 3-1	"None" vs all		"Rare" vs all		"Frequent" vs all	
				**Sens**	**Spec**	**Sens**	**Spec**	**Sens**	**Spec**

1	73.3	0	0	0.55	0.93	0.89	0.44	0.20	1

2	77.8	0	0	0.50	1	1	0.61	1	1

3	69.2	0	0	0.56	0.75	0.50	0.55	0.50	1

4	77.8	0	1	0.64	1	1	0.68	0.60	0.94

5	73.3	0	0	0.60	0.70	0.33	0.71	1	0.94

6	69.2	1	0	0.69	0.62	0.33	0.71	0.50	0.91

7	69.1	0	0	0.55	0.63	0.50	0.55	-	0.96

8	77.8	0	1	0.64	1	0.83	0.67	0.50	0.94

9	63.9	1	0	0.44	0.50	0.50	0.50	0.50	1

10	67.9	1	0	0.50	0.78	0.60	0.55	0.50	0.96

Average	71.9	3 (sum)	2 (sum)	0.57	0.79	0.65	0.60	0.59	0.96

### Layer 2

**Figure 9:**
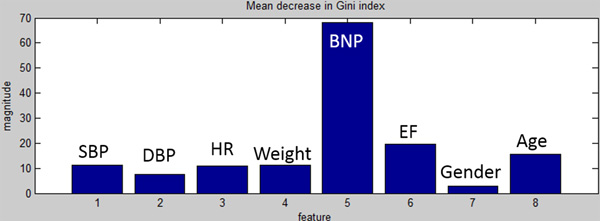
**Strong correlation between BNP (feature 5, capillary measurement) and EF (feature 6) with severity assessment**.

The aim of the DSS in Layer 2 was to classify the severity of the CHF condition as *mild, moderate *or *severe *based on data available to nurses performing home visits to the CHF patient. Similarly to the DSS of Layer 1, we evaluated the performance in this layer in terms of multiclass accuracy, sensitivity, specificity for each class vs all other classes, critical errors 1-3 and 3-1 obtained with a 10-fold cross-validation (Table 3). Notably, the average classification accuracy was 81 ± 7 % (mean ± STD), with no errors of type 1-3 and only one error of type 3-1 (CHF erroneously classified as *severe *instead of *mild*), whereas the sensitivity and specificity were 76 ± 10 % and 86 ± 7 %, respectively. Figure 9 shows the mean decrease in Gini index of the Random Forest algorithm for Layer 2, evidencing the greater sensitivity of the classifier to BNP over other features.

**Table 3:** performances of DSS of layer 2.

Fold N°	Accuracy %	N° critical error 1-3	N° critical error 3-1	"Mild" vs all		"Moderate" vs all		"Severe" vs all	
				**Sens**	**Spec**	**Sens**	**Spec**	**Sens**	**Spec**

1	81.3	0	1	0.6	1	0.8	0.73	0.7	0.8

2	71.4	0	0	0.3	1	0.83	0.47	0.8	0.9

3	79.5	0	0	0.46	1	1	0.64	0.89	1

4	77.8	0	0	0.86	0.71	0.17	0.87	0.88	0.92

5	90.0	0	0	0.83	0.93	0.88	0.83	0.83	1

6	94.9	0	0	1	0.93	0.71	1	1	0.95

7	73.8	0	0	0.88	0.50	0.33	0.92	1	1

8	83.8	0	0	0.92	0.80	0.67	0.81	0.63	1

9	80.6	0	0	0.64	0.80	0.75	0.69	1	1

10	80.3	0	0	1	0.73	0.53	1	1	0.91

Average	81.3	0	1 (sum)	0.75	0.84	0.67	0.80	0.87	0.95

### Layer 3

HRV parameters computed using the PhysioNet HRV Toolkit on 15 healthy and 15 CHF patients are reported in Table 4. Upon statistical comparison, we found that SDNN, RMSSD and pNN20 were strongly different (*p *< 0.0001) amongst CHF and healthy groups, whereas pNN50 exhibited a milder yet significant difference (*p *< 0.01). AVNN and TOTPWR were not found to be statistically different.

**Table 4:** HRV parameters computed from electrocardiogram signals collected in layer 3 on healthy and CHF patients.

HRV parameter	Healthy (N = 15)	CHF (N = 15)
AVNN	675.75 ± 115.12	642.76 ± 119.21**

SDNN	68.89 ± 9.54	21.58 ± 7.59 ***

RMSSD	40.53 ± 8.12	20.36 ± 7.56 ***

pNN20	27.86 ± 5.41	9.77 ± 7.06 ***

pNN50	4.09 ± 1.12	2.39 ± 1.79 *

TOT PWR	282.84 ± 96.15	338.87 ± 142.12**

* *p *< 0.01, ** *p *< 0.001, *** *p *< 0.0001

## Discussion

Monitoring CHF patients outside of clinical settings is known to be beneficial, primarily to reduce unnecessary readmissions [1-4]. However, adequate monitoring of CHF is not trivial due to the multifactorial nature of the disease. The distributed system proposed in this work aims at capturing multiple aspects of CHF by promoting cooperation among clinical stakeholders operating in three layers (physicians, nurses, patients) and by optimizing the trade-off between quality and quantity of acquired clinical data.

One innovative element of the proposed system is the integration of decision support systems (DSSs) appositely designed for Layers 1 (physician level) and 2 (nurse level) to facilitate development and implementation of personalized, accurate and informed strategies to maximize the outcome on each patient. Performances of DSS at both levels estimated with a 10-fold validation on a dataset of 250 CHF patients were particularly promising. The Random Forest algorithms were primarily designed to minimize the number of critical errors, particularly those of type 1-3 where DSS erroneously classifies the patient as stable instead of prone to frequent cardiac decompensations. The validation has returned only 3 errors of type 1-3 out of 64 patients with actual frequent decompensation (Layer 1), whereas the average accuracy in predicting decompensations was a promising 71.9% for a three-class classifier. Importantly, the specificity of classification into *frequent decompensation *(class 3) was quite high (96%), indicating the likelihood of the patient presenting frequent exacerbations in the future, whereas the specificity for class 1 (*no decompensations*) was found to be 79%. These results, largely driven by the strong correlation between BNP (feature 5) and decompensation events (as the Gini index in Figure 8 shows), hold potential to prevent unnecessary close monitoring of patients who are effectively managing their CHF.

DSS for Layer 2 also exhibited a good multiclass accuracy (81.3%) paired with high values of sensitivity (87%) and specificity (95%) obtained for class 3 (*severe CHF*). These results are clinically important, because they allow the nurse to identify at-risk patients with a high degree of confidence. The absence of critical errors of type 1-3 and only one of type 3-1 are also of relevance. Class 1 (*mild CHF*) was identified with good specificity (84%). In this layer, Gini Index in Figure 9 shows that the most relevant clinical parameters determining the severity of CHF are BNP (feature 5) and EF (feature 6). Hence, our results at both layers confirm previous findings that underscored BNP as a strong indicator of CHF [5].

At Layer 3, HRV analysis revealed that healthy patients exhibited measures comparable to large groups of healthy people previously reported [25,26]. In particular, we found that SDNN, RMSSD, pNN20 and pNN50 obtained from short-term EKG were significantly different between the groups (p < 0.001 or lower), which confirm previous findings on the validity of these parameters for disambiguating CHF patients from healthy controls [23].

Many artificial intelligence studies aiming at predicting the severity of CHF or the onset of deterioration have been reported using a wide variety of inputs and outputs, making a direct comparison of performances difficult. Few of these were based on machine learning techniques using HRV computed from EKG signals, similarly to the analysis performed in this work at Layer 3 [19,20,27]. Our proposed system includes novel elements in workflow modelling, management software (physician's front end), tele-monitoring and machine learning, and it yielded EKG accuracy, sensitivity and specificity results comparable with other systems [12]. Compared to a previous version of our solution described in [8,22], the results of this study showed a similar accuracy (~80%) using a larger dataset, which is particularly promising.

Yet, several issues must be resolved before further clinical testing and potential adoption in clinical settings. Most importantly, this system relies on the active participation of all CHF stakeholders and, although our proposed solution facilitates such collaboration in different aspects, it does not prescind from human willingness to engage in management that is inherently complex. Other important aspects are represented by the technical stability of the architecture and the performance of the DSSs. This architectural stability encompasses issues such as data security (both as privacy and protection against loss of data), reliability and computational optimization of software, and certification of DSSs and medical devices. This research work shows that our prototype system works well in a small-scale simulated scenario. Our future plans include improving the performance of the DSSs, especially in predicting decompensations (Layer 1), by enlarging training datasets and including additional markers of CHF reported in the literature to reflect states of inflammation, oxidative stress, neurohormonal disarray or renal injury. However, we will properly evaluate how measuring additional markers will affect the workflow, cost, and trade-off between the prognostic power of the clinical measures and the ability of the system to monitor them frequently. Also, we will continue to investigate both the importance of a properly trained medical staff and the role of the patient and his/her ability to effectively perform self-management at home by considering training and factors affecting adherence to the protocols.

## Conclusion

In this paper, we proposed a collaborative system for the comprehensive care of congestive heart failure, beginning in the hospital at the first admission (or first diagnosis made during an office visit) and continuing through home monitoring. This approach is in line with the increasingly popular Chronic Care Model.

The proposed system consists of three layers of monitoring: Layer 1 in the hospital or cardiologist's office, Layer 2 with nurses vising the patient at home, and Layer 3 with self-monitoring by the patient. The prognostic value of the CHF parameters measured at each level decreases from Layer 1 to Layer 3, but the measurement frequency increases to establish a paradigm in which the patient is constantly monitored. Since CHF is a multi-parametric and multifactorial disease, all available information collected at all layers is accounted for in automated decision support systems (DSSs) based on machine learning techniques, and is easily accessible by all stakeholders to facilitate decision-making. The performance of DSSs trained with first-visit datasets showed promising accuracy, sensitivity and specificity of CHF classification in three classes (Layer 1: absent, rare, frequent decompensation; Layer 2: mild, moderate, severe CHF) and confirmed results of our previous work. We found strong correlation between CHF markers (BNP and EF) and disease severity and frequency of decompensation, which confirm literature findings. We also found that heart rate variability (HRV) computed on EKG self-acquired by patients with devices for home monitoring were significantly different in CHF and healthy cohorts.

## Competing interests

There are no competing interests to declare.

## Authors' contributions

G. Guidi, L. Pollonini and E. Iadanza contributed equally to this paper, both in performing the research and in writing the manuscript. C. C. Dacso is the principal investigator and co-developer of the BlueBox/BluScale systems.

## Declarations

Research and publication costs for this work were funded by the Department of Information Engineering at the Università degli Studi di Firenze and the Abramson Center for the Future of Health at the University of Houston. The authors wish to thank Kara K. McArthur for editing the manuscript.

This article has been published as part of *BMC Medical Informatics and Decision Making *Volume 15 Supplement 3, 2015: Multidimensional, multidiscipline and shared decision making in healthcare and eHealth. The full contents of the supplement are available online at http://www.biomedcentral.com/bmcmedinformdecismak/supplements/15/S3.
